# Smarter Scanning: Reducing Unnecessary Magnetic Resonance Cholangiopancreatography (MRCP) in Low-Risk Gallstone Patients

**DOI:** 10.7759/cureus.89959

**Published:** 2025-08-13

**Authors:** Najeeb Aftab, Khalid Sohail, Nicole Handy, Abbie Evans, Helen Rotherforth

**Affiliations:** 1 General Surgery, Mid Yorkshire Teaching NHS Trust, Wakefield, GBR; 2 Knowledge and Library Services, Mid Yorkshire Teaching NHS Trust, Wakefield, GBR

**Keywords:** gallstone disease (gsd), liver function tests (lfts), magnetic resonance cholangiopancreatography (mrcp), ultrasonography (usg), upper gastrointestinal surgery

## Abstract

Background

Gallstone disease is a common condition that often requires imaging to exclude choledocholithiasis. Magnetic resonance cholangiopancreatography (MRCP) is a highly accurate but costly scan, increasingly used in low- to moderate-risk patients where its diagnostic yield may be low.

Objective

This audit evaluated the diagnostic yield of MRCP in low- to moderate-risk gallstone patients and assessed the predictive value of liver function tests (LFTs) and ultrasound (USS) findings to develop a smarter referral approach.

Methods

A retrospective audit was conducted at a single NHS Trust from January to December 2024. Data on MRCP outcomes, pre-scan LFTs (bilirubin, alkaline phosphatase (ALP)), and USS common bile duct (CBD) diameter were analyzed using chi-squared tests. A composite score (MRCP-RS) combining key predictors was explored to guide smarter MRCP referrals.

Results

Among 329 MRCPs, 42.2% were normal. Elevated bilirubin and ALP showed no significant association with abnormal MRCPs (p=1.00 and p=0.61). Dilated CBD on USS had limited predictive value (p=0.82). The MRCP-RS composite score demonstrated a trend of increasing abnormal MRCP rates with higher scores but modest discriminative ability. Avoidable normal MRCPs incurred an estimated annual cost of £38,000-65,000.

Conclusion

Routine use of MRCP in low-risk gallstone patients leads to unnecessary imaging and costs. Neither LFTs nor USS alone is a reliable predictor. A combined approach using a simple composite score may improve referral decisions. Adoption of smarter referral tools and re-audit post-implementation are recommended.

## Introduction

Gallstone disease is one of those stubbornly common conditions in modern healthcare, affecting around 10-15% of adults in Western nations and up to 20% globally [1]. For many, the journey ends with a straightforward laparoscopic cholecystectomy [1,2], but for a significant number, the path isn't so clear-cut, particularly when doctors are faced with a familiar dilemma: could there be stones lurking in the common bile duct (CBD)? That question becomes trickier when the patient is only at low to moderate risk.

Enter magnetic resonance cholangiopancreatography (MRCP), a non-invasive, high-resolution scan that's become the go-to for detecting choledocholithiasis [2,3]. It's precise, safe, and reassuringly thorough. But here's the catch: it's also expensive, time-consuming, and increasingly overused, especially in patients who might not need it [4,5]. The broader picture of gallstone disease includes everything from asymptomatic stones to full-blown pancreatitis or cholangitis. When there's suspicion of CBD stones, clinicians often rely on liver function tests (LFTs) like bilirubin, alkaline phosphatase (ALP), and alanine aminotransferase (ALT), along with ultrasound scans of the bile ducts, to help them decide whether an MRCP is warranted [2,3]. ERCP, while both diagnostic and therapeutic, carries more risk and is generally reserved for confirmed cases [4].

Despite the 2014 National Institute for Health and Care Excellence (NICE) Clinical Guideline CG188 [2], which offers a general roadmap for managing gallstone disease, there's still no agreed national cut-off or algorithm for ordering MRCP in low-risk patients. That leaves plenty of room for variability, with some clinicians opting for early imaging and others taking a watch-and-wait approach. This inconsistency is well documented in national surveys like the ALiCE study [4].

Meanwhile, studies like the ongoing Sunflower Trial are digging into whether we might be better off skipping MRCP in select patients altogether. Could expectant management be just as safe and far more efficient [5]? Until those answers are clear, many Trusts operate in a grey zone without formal MRCP referral policies.

And the cost? It's no small matter. At £275-470 per scan [6], plus the ripple effect of downstream procedures and hospital stays, MRCP overuse puts real strain on NHS resources. Our own audit tells the story even more plainly: 42.2% of MRCPs we performed for gallstone-related reasons came back completely normal, despite every patient ticking at least one Sunflower Study inclusion box. That's a lot of expensive reassurance.

On the horizon, artificial intelligence (AI) and machine learning (ML) models are making waves. These tools (YOLOv5, random forest) have shown they can outperform traditional risk stratification in predicting choledocholithiasis, offering a glimpse into a future of smarter, leaner decision-making [7-10].

In this study, we examine our own Trust's MRCP usage patterns. We look at diagnostic yield, the role of LFTs and ultrasound (USS) in predicting outcomes, and where we might be overreaching. The goal? To build a case for more consistent, cost-effective referral practices, possibly even powered by AI.

Given these factors, the present study aimed to evaluate MRCP ordering practices within a single UK Trust by assessing the diagnostic yield of scans in low- to moderate-risk gallstone patients, determining the independent predictive value of key biochemical markers (bilirubin, ALP) and radiological findings (CBD diameter), developing and testing a simple composite risk score (MRCP-RS) to improve referral accuracy, and estimating the potential cost savings from reducing unnecessary MRCPs. While AI-based approaches were not implemented in this audit, emerging evidence suggests they may further refine MRCP referral pathways [11], and our findings provide baseline data for such future work.

## Materials and methods

Study design and setting

This was a retrospective clinical audit conducted within the General Surgery Department across three hospital sites of the Mid Yorkshire Teaching NHS Trust: Pinderfields Hospital (Wakefield), Dewsbury and District Hospital, and Pontefract Hospital. The audit reviewed MRCP requests made for gallstone-related indications in adult patients over a 12-month period, from January 1 to December 31, 2024.

Inclusion criteria

All adult patients admitted under the General Surgery Department between January and December 2024 who underwent MRCP for suspected choledocholithiasis were included. Patients were classified as low to moderate risk according to NICE CG188 criteria (absence of clinical jaundice, cholangitis, or pancreatitis), with the additional requirement that pre-scan biochemical markers met at least one of the following thresholds: bilirubin <50 µmol/L and ALP <3× upper limit of normal. These supplementary thresholds were selected to align with the Sunflower Trial framework and prior predictive studies [5].

Exclusion criteria

Patients were excluded if they presented with a known diagnosis of choledocholithiasis at admission, were referred from hepatobiliary specialist centers, or underwent MRCP for non-gallstone-related indications such as suspected malignancy or chronic liver disease. Cases with incomplete clinical, biochemical, or imaging data were also excluded from analysis.

Data collection and variables

Data were collected retrospectively using a structured audit proforma developed and piloted prior to implementation. Data were extracted from the Trust's Patient Pathway Manager (PPM), Picture Archiving and Communication System (PACS; Sectra), and Integrated Clinical Environment (ICE) laboratory system. For patients with multiple LFT results, the value taken closest in time to the MRCP (within 24 hours before imaging) was used. For each patient, the audit recorded key biochemical markers, including LFTs (bilirubin, ALP, and ALT), along with USS findings such as the presence of gallstones and measurement of CBD diameter [12]. MRCP reports were reviewed and classified as either normal or abnormal, with abnormal findings including the presence of CBD stones, strictures, or ductal dilatation [13]. Where applicable, ERCP outcomes were also noted to assess the concordance between diagnostic imaging and therapeutic intervention. Additionally, the audit captured whether patients proceeded to surgery or other interventional procedures following imaging.

Statistical analysis

For statistical testing, abnormal liver function was defined as bilirubin >36 µmol/L and ALP >250 U/L. These thresholds were selected based on ranges supported in predictive modelling and imaging literature [12-14] and align with cut-offs frequently used in prior studies assessing choledocholithiasis risk. CBD dilatation was defined as >7 mm on USS, consistent with published imaging criteria [13]. 

Composite score development (MRCP-RS)

A simple composite referral score, the MRCP-RS, was developed during data analysis as an exploratory triage tool to combine common biochemical and radiological referral triggers into a single metric. The three components were chosen based on established predictive value in the literature and alignment with the statistical thresholds pre-specified in this study: bilirubin >36 µmol/L, ALP >250 U/L, and CBD diameter >7 mm on USS [12-14]. Each parameter was assigned one point if the threshold was met, producing a total score range of 0-3. The aim was to evaluate whether higher scores correlated with higher rates of abnormal MRCP findings in this audit cohort.

Data analysis tools

Data analysis was carried out using Microsoft Excel (Version 2506, Microsoft Corporation, Redmond, Washington, United States). Descriptive statistics were calculated to determine frequencies, percentages, and mean values for key variables such as bilirubin, ALP, and CBD diameter. Chi-squared tests were applied to evaluate the association between LFT abnormalities, USS findings, and MRCP outcomes. Graphs and tables were generated within Excel to visualize the diagnostic yield and predictive trends. Generative AI tools (OpenAI ChatGPT, 2025 version, San Francisco, California, United States) were used solely to improve the clarity and flow of the Results narrative, including summarizing already-completed statistical outputs and refining wording. All numerical calculations, p-values, and statistical interpretations were performed exclusively by the authors and manually verified for accuracy and reliability.

Outcomes measured

The primary outcome was the diagnostic yield of MRCP in low- to moderate-risk gallstone patients. Secondary outcomes included the association between MRCP findings and key pre-imaging indicators (bilirubin, ALP, and CBD diameter on USS), the rate and outcome of endoscopic retrograde cholangiopancreatography (ERCP) following MRCP, and the performance of the MRCP-RS composite score described above.

## Results

**Figure 1:**
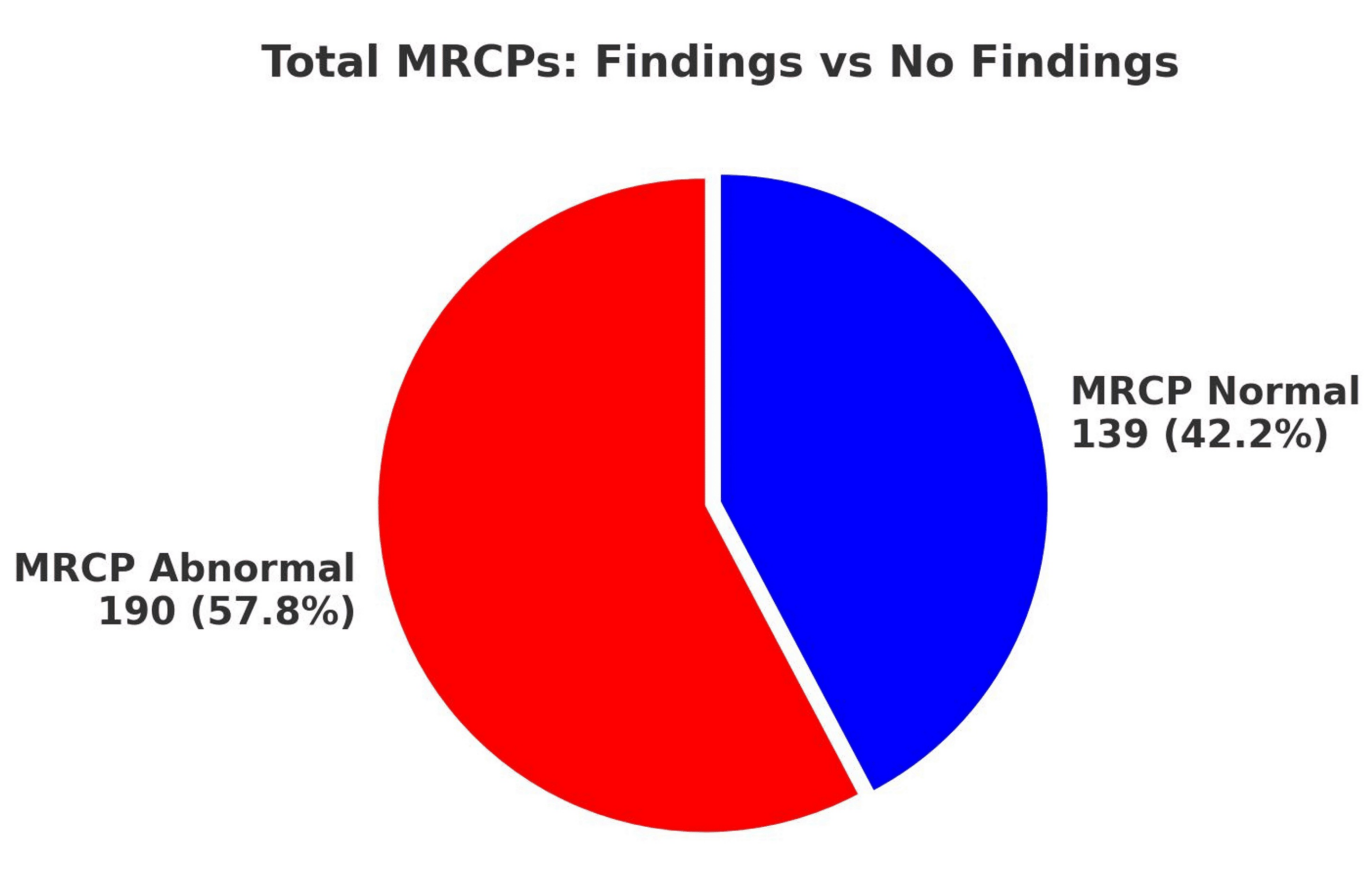
MRCP burden Distribution of MRCP outcomes (normal vs. abnormal) in gallstone patients (n=329). Abnormal MRCP findings included choledocholithiasis, strictures, or biliary dilatation. Percentages represent the proportion of all MRCPs performed. MRCP: magnetic resonance cholangiopancreatography

A total of 329 MRCPs were performed within the audit period. Of these, 190 MRCPs (57.8%) revealed abnormal findings, while 139 MRCPs (42.2%) were normal. Notably, all of these patients met at least one of the Sunflower Study inclusion criteria. MRCP abnormalities encompassed findings such as stones, strictures, and CBD dilatation. Notably, despite the presence of significant clinical indicators leading to the MRCP request, a substantial portion (42.2%) yielded normal results, potentially due to stone passage prior to imaging (Figure 1).

Statistical association of LFTs with MRCP findings

**Figure 2:**
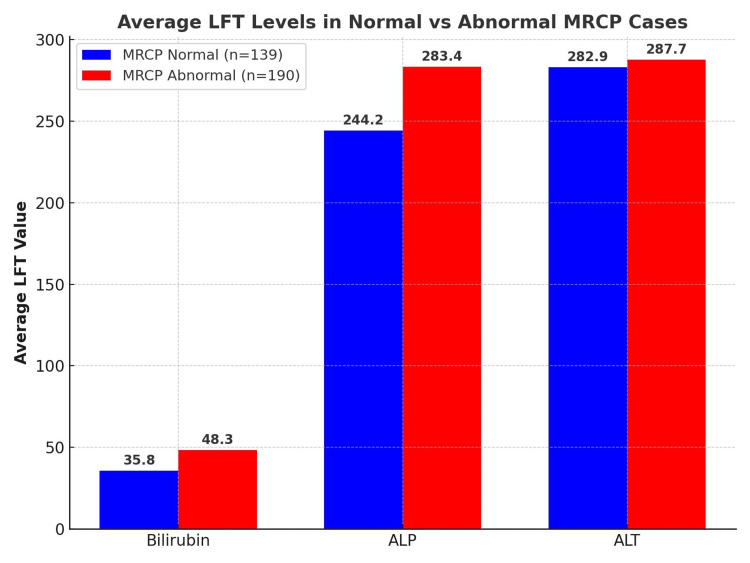
Average LFT levels in normal vs. abnormal MRCP cases This bar chart compares the mean values of key LFTs, namely, bilirubin, ALP, and ALT, between patients with normal MRCP results (n=139) and those with abnormal MRCP findings (n=190). Each bar displays the average value for that group, with MRCP abnormal cases shown in red and MRCP normal cases shown in blue. While elevated bilirubin (>36 μmol/L) and ALP (>250 U/L) were numerically higher in the abnormal MRCP group, these differences did not reach statistical significance (p=1.00 for bilirubin; p=0.61 for ALP). LFT: liver function test; MRCP: magnetic resonance cholangiopancreatography; ALP: alkaline phosphatase; ALT: alanine aminotransferase

A chi-squared test was conducted to evaluate whether LFT abnormalities, as defined in the Methods, predicted abnormal MRCP outcomes. No statistically significant association was found between elevated bilirubin and abnormal MRCP findings (p=1.00) or between elevated ALP and abnormal MRCP findings (p=0.61). This suggests that, although LFT derangements are frequently used as referral triggers, they did not independently predict abnormal MRCP results in this cohort (Table 1 and Figure 2).

**Table 1:** LFT outcomes LFT: liver function test; MRCP: magnetic resonance cholangiopancreatography; ALP: alkaline phosphatase

LFT marker	MRCP result	High LFT	Normal LFT
Bilirubin >36	Abnormal MRCP	86	104
	Normal MRCP	63	76
ALP >250	Abnormal MRCP	72	118
	Normal MRCP	48	91

Ultrasound findings vs. MRCP statistical analysis

**Figure 3:**
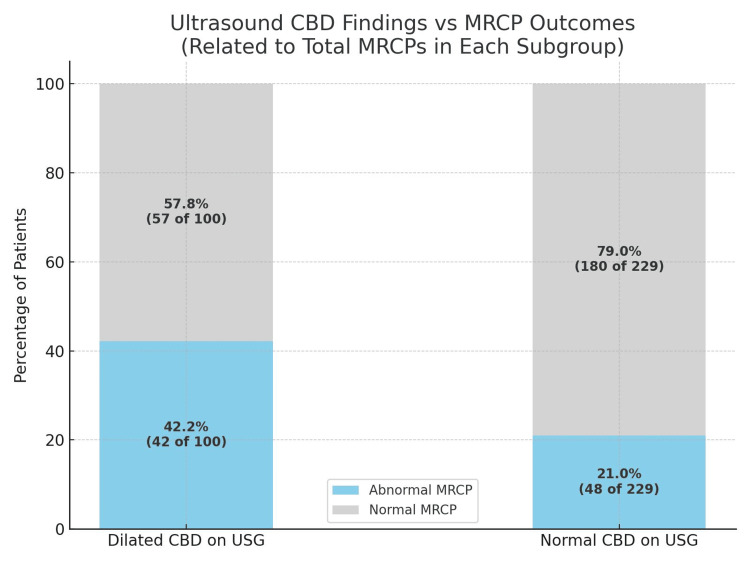
Ultrasound CBD findings vs. MRCP outcomes This bar chart compares MRCP outcomes between patients with dilated CBD on USG (n=100) and those with normal CBD diameter (n=229). Each bar segment shows the number of patients (N) and percentage (%) of abnormal and normal MRCP findings within each subgroup. Among patients with dilated CBD on USG, 42 (42.2%) had abnormal MRCP findings, while 57 (57.8%) had normal MRCPs. In the normal CBD group, 48 (21%) had abnormal MRCPs, and 180 (79%) had normal findings. This highlights the limited predictive value of USG findings alone for MRCP outcomes. MRCP: magnetic resonance cholangiopancreatography; CBD: common bile duct; USG: ultrasound

Ultrasound assessment of the CBD showed a numerical trend toward predicting abnormal MRCP findings. Among patients with a dilated CBD on ultrasound (>7 mm), 42% had abnormal MRCPs, compared to 21% in those with a normal CBD. However, a chi-squared test confirmed that this difference was not statistically significant (p=0.82). Sensitivity and specificity were modest at 42% and 60%, respectively, indicating that USS alone has limited predictive value and should be integrated with biochemical and clinical indicators (Table 2 and Figure 3).

**Table 2:** Ultrasound results MRCP: magnetic resonance cholangiopancreatography; CBD: common bile duct

MRCP result	CBD dilated (n=100)	CBD normal (n=229)
Abnormal MRCP	42	48
Normal MRCP	58	181

**Figure 4:**
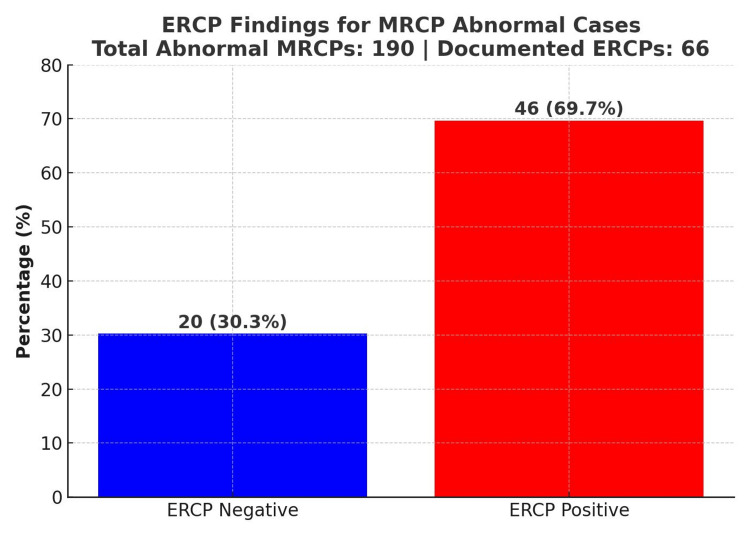
ERCP outcomes following abnormal MRCP findings This bar chart illustrates the outcomes of ERCP in patients who had abnormal MRCP findings (n=190). Of these, 66 patients underwent ERCP. Among them, 46 patients (69.7%) had positive ERCP findings, confirming biliary pathology (e.g., choledocholithiasis or strictures), while 20 patients (30.3%) had negative ERCP results, indicating that MRCP may overestimate pathology in certain cases. Each bar displays both the number of patients (N) and the percentage (%) relative to total documented ERCPs. ERCP: endoscopic retrograde cholangiopancreatography; MRCP: magnetic resonance cholangiopancreatography

In the correlation between MRCP and ERCP outcomes, 69.7% of patients with abnormal MRCP findings proceeded to have positive ERCPs, confirming biliary pathology. However, 30.3% had negative ERCPs, indicating that MRCP may sometimes overestimate pathology (Figure 4). This suggests the need for improved pre-MRCP assessment protocols.

Composite score for MRCP referral MRCP-RS score: a triage tool

**Figure 5:**
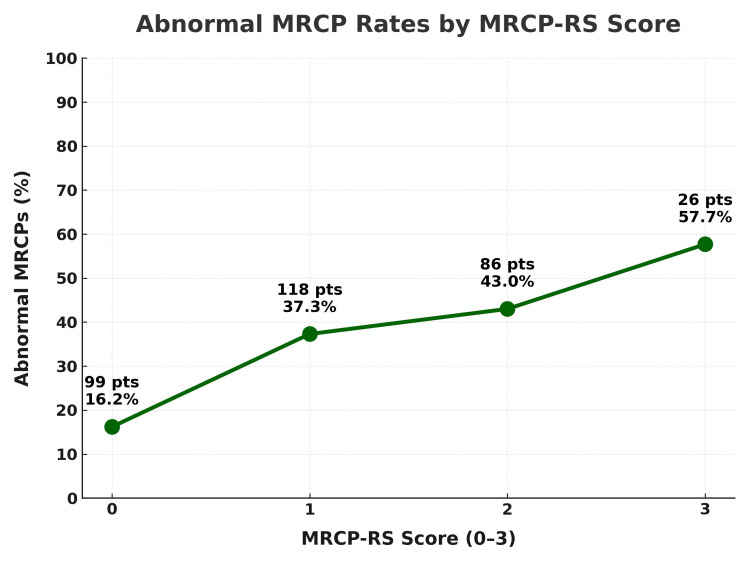
Abnormal MRCP rates by MRCP-RS score This line chart illustrates the relationship between the MRCP-RS composite score (range 0-3) and the percentage of abnormal MRCP findings among the audited patients (n=329). The MRCP-RS score incorporates three predictors: bilirubin >36 μmol/L, ALP >250 U/L, and CBD diameter >7 mm. The number of patients (N) and the corresponding percentage of abnormal MRCPs are displayed for each score group. Abnormal MRCP rates rose from 16.2% (16/99) at score 0 to 57.7% (15/26) at score 3, demonstrating that higher scores were associated with increased likelihood of abnormal findings. While the overall discriminative power of the score was modest, the trend supports combining biochemical and imaging markers to refine MRCP referral decisions. MRCP: magnetic resonance cholangiopancreatography; ALP: alkaline phosphatase; CBD: common bile duct

As described in the Methods, we evaluated the MRCP-RS, an exploratory additive score based on three common referral triggers. In our cohort, abnormal MRCP rates were lowest in patients scoring 0 and generally higher with increasing scores, indicating that meeting more criteria was associated with a greater likelihood of significant pathology. Although the discriminative ability was modest (AUC=0.57), the overall trend supports the value of combining biochemical and radiological predictors rather than relying on a single trigger. This simple tool could function as a gatekeeper in settings without algorithmic decision support, pending the development of national stratification guidelines or AI-enhanced referral systems (Table 3 and Figure 5).

**Table 3:** Components of MRCP-RS (composite score) ALP: alkaline phosphatase; CBD: common bile duct; USS: ultrasound

Criterion	Score assigned	Patients meeting the criterion
Bilirubin >36 µmol/L	1	36
ALP >250 U/L	1	28
CBD >7 mm (USS)	1	73

## Discussion

This audit throws a spotlight on a common but often overlooked habit in surgical diagnostics, the generous use of MRCP in patients who might not really need it. Despite following the inclusion criteria laid out in the Sunflower framework [5], 42.2% of MRCPs in our cohort came back completely normal. That's a lot of scans with no actionable findings, and it raises fair questions about how we're using this powerful (and expensive) tool.

Let's be clear: MRCP is fantastic when used appropriately. It's non-invasive and highly sensitive and gives us gorgeous images of the biliary tree. But our findings suggest that when used indiscriminately, especially in low-yield cases, it becomes more of a reflex than a reasoned choice. Markers like slightly raised bilirubin or a CBD that looks "maybe a bit wide" on USS often tip the scale toward ordering an MRCP [15]. And yet, both LFTs and USS in our data showed poor correlation with actual pathology. Bilirubin and ALP did not reach statistical significance, and USS CBD dilation likewise showed only a numerical trend without statistical significance, reinforcing its limited predictive value in isolation [15].

More to the point, when we subjected our data to formal statistical testing, the case for restraint only grew stronger. A chi-squared analysis revealed no significant association between elevated bilirubin or ALP levels and abnormal MRCP results (p=1.00 and p=0.61, respectively). Even patients with "concerning" biochemistry often had normal imaging. USS didn't fare much better. CBD dilation over 7 mm showed a numerical association with abnormal MRCP findings but was not statistically significant (p=0.82) and had limited predictive utility in isolation. These aren't the kind of numbers that inspire clinical confidence. In short, the two most common MRCP referral triggers, LFTs and USS, may look convincing at the bedside but don't hold up statistically when tested.

Interestingly, a 2017 institutional review asked a very relevant question: Is MRCP actually changing what we do for patients [16]? In their analysis of suspected choledocholithiasis cases, they found that MRCP often didn't shift the treatment plan. Many patients still ended up having ERCP or surgery, regardless of what the scan showed [16]. In low- to intermediate-risk cases, MRCP sometimes just confirmed what clinical judgment had already suspected, offering reassurance but not necessarily altering the course. The authors raised a valid concern: Are we leaning too heavily on imaging when history, labs, and USS might already be enough? Their findings echo ours and add weight to the argument for being more selective with who needs an MRCP.

Then there's the money. At £275-470 per scan [6], the 139 normal MRCPs we clocked up translate into an avoidable bill somewhere between £38,000 and £65,000. And that's just the tip of the iceberg; it doesn't count the knock-on costs of extra ERCPs, delays to theatre, or longer hospital stays. In fact, newer studies suggest that MRCP may not be the time-saver we think it is. One 2025 study found no significant difference in length of stay between MRCP-first, ERCP-first, or indicators of compromise (IOC)-based pathways [17]. Another from 2015 showed that starting with surgery and IOC might actually get patients out of the hospital sooner [18].

And let's not forget the clinical risks. MRCP isn't perfect. False positives can lead to unnecessary ERCPs (and all the risks that come with them, like pancreatitis and bleeding) [19] (Figure 4), while false negatives can delay treatment when patients seem fine but still harbour stones. Over time, this feeds into what the ALiCE survey called clinical inertia, ordering an MRCP "just in case", even when evidence suggests we should wait or watch [4].

In light of this, we trialled a more nuanced approach: a simple composite referral score, the MRCP-RS, combining bilirubin >36 µmol/L, ALP >250 U/L, and CBD diameter >7 mm [12,13]. It's not groundbreaking, but it does paint a clearer picture (Figure 5). Abnormal MRCP rates rose from 16.2% (16/99) with a score of 0 to 57.7% (15/26) when all three criteria were met. The tool's overall discriminative power was modest (AUC=0.57), but the consistent upward trend supports the principle that combining predictors is more informative than relying on any single biochemical or radiological trigger. It reinforces the idea that no single trigger should tip the scale, but that patterns matter. A scoring system, even a simple one, could serve as a gatekeeper, helping us pause, assess, and avoid reaching for MRCP on autopilot.

So where do we go from here? Enter: AI. Recent reviews show that ML models can actually outperform or rival guideline-based stratification [7-10] in predicting choledocholithiasis, especially in picking out the people who don't need a scan [7-10]. These models use a mix of routine data, bilirubin, lipase, CBD size, and clinical features, and turn them into smarter, faster triage tools. In some studies, they've helped cut down imaging rates without sacrificing accuracy or safety [10]. And while they're not perfect yet, they're getting there fast.

What we need now is a local rethink. We're not calling for MRCP to be shelved, far from it. But it should be treated with the respect it deserves: a valuable investigation reserved for the right patients. We propose a composite referral tool that blends LFTs, USS findings, symptoms, and maybe even AI predictions, instead of leaning on any single flag. And as we wait for the results of the Sunflower Trial [5], there's no reason we can't start tightening up our own protocols at the Trust level.

In the end, this is about doing better with what we already have. MRCP is brilliant, but only when it's needed. Smarter use means less delay, less cost, and better outcomes. That's the kind of scan worth ordering.

Limitations

This audit has several limitations that may have influenced the findings. The retrospective design meant we relied on existing clinical records, which were sometimes incomplete or inconsistently documented, leading to the possibility of data gaps or bias. There was also variation in how ultrasound scans were performed and reported, particularly when measuring the CBD, which could have affected the correlation with MRCP results. The biochemical thresholds used for bilirubin (>36 µmol/L) and ALP (>250 U/L) were selected pragmatically, based on local practice and values supported in previous studies, but were not validated within this dataset or in a prospective setting; this may limit the generalizability of our findings. As this was a single-center study, the results may not reflect practices in other hospitals or regions. We also did not include a control group or assess long-term patient outcomes, so the impact of MRCP findings on overall management cannot be fully evaluated. Finally, although we used chi-squared testing, the sample size and the number of borderline values may have limited our ability to detect more subtle, clinically relevant associations.

## Conclusions

This audit underscores a critical issue in current surgical practice: the liberal use of MRCP in low- to moderate-risk gallstone patients, often based on weak predictors such as mildly abnormal LFTs or borderline CBD dilatation. Despite aligning with existing referral frameworks like the Sunflower inclusion criteria, over 40% of MRCPs yielded normal findings, highlighting both clinical inefficiency and a significant cost burden.

Our data confirm that no single parameter, biochemical or radiological, offers sufficient predictive value in isolation. Instead, a composite approach, such as the proposed MRCP-RS score, may help refine clinical decision-making by integrating bilirubin levels, ALP, and USS findings. While not a definitive rule, this tool offers a practical step toward more consistent and evidence-based referral practices. Future re-audit within three months will be considered to assess whether refined MRCP referral criteria translate into improved diagnostic yield and cost-effectiveness.

At present, there is no nationally agreed guideline for MRCP referral in this patient group, leading to variation and potential overuse. As the results of the Sunflower Trial are awaited, our findings support immediate local efforts to adopt structured referral criteria, enhance clinician awareness, and reduce unnecessary imaging, ultimately improving both patient care and resource stewardship.

## References

[1] (2019). Gallstone disease: diagnosis and management. October.

[2] (2018). 2018 Surveillance of Gallstone Disease: Diagnosis and Management (NICE Clinical Guidance CG188). https://www.ncbi.nlm.nih.gov/books/NBK550940/.

[3] (2016). Commissioning Guide: Gallstone Disease. https://www.rcseng.ac.uk/-/media/files/rcs/standards-and-research/commissioning/gallstone-disease-commissioning-guide-for-republication.pdf.

[4] Tanase A, Dhanda A, Cramp M, Streeter A, Aroori S (2022). A UK survey on variation in the practice of management of choledocholithiasis and laparoscopic common bile duct exploration (ALiCE Survey). Surg Endosc.

[5] Clout M, Blazeby J, Rogers C (2021). Randomised controlled trial to establish the clinical and cost-effectiveness of expectant management versus preoperative imaging with magnetic resonance cholangiopancreatography in patients with symptomatic gallbladder disease undergoing laparoscopic cholecystectomy at low or moderate risk of common bile duct stones (the Sunflower Study): a study protocol. BMJ Open.

[6] British Heart Foundation (2022). Guidelines for costing clinical research imaging scans. Clinical Research Imaging Scan Costing Guidelines. October.

[7] Blum J, Wood L, Turner R (2025). Artificial intelligence in the detection of choledocholithiasis: a systematic review. HPB.

[8] Blum J, Hunn S, Smith J, Chan FY, Turner R (2024). Using artificial intelligence to predict choledocholithiasis: can machine learning models abate the use of MRCP in patients with biliary dysfunction?. ANZ J Surg.

[9] Luo B, Li Z, Zhang K (2024). Using deep learning models in magnetic resonance cholangiopancreatography images to diagnose common bile duct stones. Scand J Gastroenterol.

[10] Huerta-Reyna R, Guevara-Torres L, Martínez-Jiménez MA, Armas-Zarate F, Aguilar-García J, Waldo-Hernández LI, Martínez-Martínez MU (2024). Development and validation of a predictive model for choledocholithiasis. World J Surg.

[11] Doherty G, Manktelow M, Skelly B, Gillespie P, Bjourson AJ, Watterson S (2022). The need for standardizing diagnosis, treatment and clinical care of cholecystitis and biliary colic in gallbladder disease. Medicina (Kaunas).

[12] Peng WK, Sheikh Z, Paterson-Brown S, Nixon SJ (2005). Role of liver function tests in predicting common bile duct stones in acute calculous cholecystitis. Br J Surg.

[13] Perret RS, Sloop GD, Borne JA (2000). Common bile duct measurements in an elderly population. J Ultrasound Med.

[14] Mohammad H, Achinge GI, Eke BA (2013). Sonographic assessment of common bile duct diameter among adults in North Central Nigeria. IOSR J Dent Med Sci.

[15] Samara O, Azzam MI, Alshrouf MA (2022). Diagnostic accuracy of ultrasonography compared with magnetic resonance cholangiopancreatography in the detection of choledocholithiasis. J Clin Ultrasound.

[16] Badger WR, Borgert AJ, Kallies KJ, Kothari SN (2017). Utility of MRCP in clinical decision making of suspected choledocholithiasis: an institutional analysis and literature review. Am J Surg.

[17] Kelley JK, Mormol J, Reiber M (2025). Cost-benefit analysis of various management algorithms for suspected choledocholithiasis. Am Surg.

[18] Lin C, Collins JN, Britt RC, Britt LD (2015). Initial cholecystectomy with cholangiography decreases length of stay compared to preoperative MRCP or ERCP in the management of choledocholithiasis. Am Surg.

[19] Hjartarson JH, Hannesson P, Sverrisson I, Blöndal S, Ívarsson B, Björnsson ES (2016). The value of magnetic resonance cholangiopancreatography for the exclusion of choledocholithiasis. Scand J Gastroenterol.

